# Moderate Caloric Restriction during Gestation in Rats Alters Adipose Tissue Sympathetic Innervation and Later Adiposity in Offspring

**DOI:** 10.1371/journal.pone.0017313

**Published:** 2011-02-18

**Authors:** Ana Paula García, Mariona Palou, Juana Sánchez, Teresa Priego, Andreu Palou, Catalina Picó

**Affiliations:** Laboratory of Molecular Biology, Nutrition and Biotechnology (Nutrigenomics), University of Balearic Islands and CIBER Fisiopatología de la Obesidad y Nutrición (CIBEROBN), Palma de Mallorca, Spain; University of Córdoba, Spain

## Abstract

Maternal prenatal undernutrition predisposes offspring to higher adiposity in adulthood. Mechanisms involved in these programming effects, apart from those described in central nervous system development, have not been established. Here we aimed to evaluate whether moderate caloric restriction during early pregnancy in rats affects white adipose tissue (WAT) sympathetic innervation in the offspring, and its relationship with adiposity development. For this purpose, inguinal and retroperitoneal WAT (iWAT and rpWAT, respectively) were analyzed in male and female offspring of control and 20% caloric-restricted (from 1–12 d of pregnancy) (CR) dams. Body weight (BW), the weight, DNA-content, morphological features and the immunoreactive tyrosine hydroxylase and Neuropeptide Y area (TH+ and NPY+ respectively, performed by immunohistochemistry) of both fat depots, were studied at 25 d and 6 m of age, the latter after 2 m exposure to high fat diet. At 6 m of life, CR males but not females, exhibited greater BW, and greater weight and total DNA-content in iWAT, without changes in adipocytes size, suggesting the development of hyperplasia in this depot. However, in rpWAT, CR males but not females, showed larger adipocyte diameter, with no changes in DNA-content, suggesting the development of hypertrophy. These parameters were not different between control and CR animals at the age of 25 d. In iWAT, both at 25 d and 6 m, CR males but not females, showed lower TH^+^ and NPY^+^, suggesting lower sympathetic innervation in CR males compared to control males. In rpWAT, at 6 m but not at 25 d, CR males but not females, showed lower TH^+^ and NPY^+^. Thus, the effects of caloric restriction during gestation on later adiposity and on the differences in the adult phenotype between internal and subcutaneous fat depots in the male offspring may be associated in part with specific alterations in sympathetic innervation, which may impact on WAT architecture.

## Introduction

A crescent body of evidence has demonstrated that hormonal, metabolic and nutritional disturbances at a critical, sensitive period of early life may determine the propensity to the development of obesity and its related metabolic pathologies [1], [2], [3]. In particular, maternal prenatal undernutrition has been described to have long term consequences on offspring metabolic energy regulatory systems [4], [5], increasing the susceptibility to the development of excessive adiposity, particularly when exposed postnatally to a high fat (HF) diet [4], [5], [6].

Perturbations in central structures of the nervous system involved in the control of food intake and energy expenditure have been recently proposed to account for the lasting effect of these perinatal conditions [7], [8]. In particular, the offspring of rats exposed to 20% caloric restriction during gestation exhibited fewer neuropeptide Y- and α-melanocyte-stimulating hormone-neurons and fewer total cells in arcuate nucleus [7]. However, apart from the effects on the central nervous system (CNS), there is little information on possible programming effects of perinatal conditions on the peripheral nervous system structures involved in the control of energy metabolism and adipose tissue growth. Of interest, long lasting effects of maternal undernutrition have been observed on enteric sympathetic nervous system (SNS) in the offspring; 50% maternal caloric restriction during the last two weeks of pregnancy reduced enteric sympathetic innervation [9], and also norepinephrine (NE) levels in the coeliac-superior mesenteric ganglion complex and the diameter of its neurons [10]. These findings suggest that the development of sympathetic innervation could be affected by nutritional disturbances during critical periods of development and be responsible for some of the lasting effects of these conditions.

SNS innervates subcutaneous and intraperitoneal white adipose tissue (WAT) depots. The best established role for this sympathetic innervation in WAT is the stimulation of lipid mobilization [11], [12]. These effects are mediated principally by NE, the main neurotransmitter released by the terminals of postganglionic sympathetic neurons on WAT [12], which exerts its effects on energy metabolism principally through adrenergic receptors (AR) expressed in adipocytes. It has been observed that the acute administration of a β3-AR agonist increases oxygen consumption, insulin secretion, and reduces food intake [13], and in the adipocyte it stimulates lipolysis [14].

Besides its effects on adipocyte metabolism, sympathetic innervation also plays an important role in the control of adipocyte proliferation [15]. In fact, it has been observed that experimental denervation of adipose tissue is accompanied by increased total fat cell number in the inguinal WAT (iWAT) [16], [17], [18]. Supporting this function of SNS in WAT proliferation are the findings that NE inhibits the natural proliferation of pre-adipocytes in primary culture and in Chinese hamster ovary/K1 cells expressing human β3-AR [19], [20].

In addition to NE, neuropeptide Y (NPY) has also been recently described to be involved in adipocyte proliferation. NPY is synthesized in neural tissue of the central and peripheral nervous systems, and has a number of important functions besides regulating appetite and energy homeostasis [21]. At periphery, NPY is colocalized with NE and released from sympathetic nerve terminals on sympathetic stimulation [22], [23]. However, unlike NE, NPY stimulates adipocyte proliferation [24], [25].

In the present study we aimed to evaluate whether moderate maternal caloric restriction (20%) during the first 12 days of gestation in rats was able to produce early effects in the offspring on WAT sympathetic innervation, thus affecting NE and NPY content in nerve terminals, and also to study its relationship with adiposity development when animals are exposed to a dietary stressor such as HF diet in adult life. To analyse fat depot- and gender-specific effects, two WAT depots, one internal (retroperitoneal) and one subcutaneous (inguinal), were analysed in both male and female animals when they were 25 d old (an age at which animals had just begun to feed independently but still showed no alteration in body weight), and at the age of 6 m, after a period of 2 m of HF diet exposure.

## Materials and Methods

### Animals and Experimental Design

The animal protocol followed in this study was reviewed and approved by the Bioethical Committee of the University of the Balearic Islands (resolution number 1798 of February 18^th^, 2009) and guidelines for the use and care of laboratory animals of the University were followed.

The study was performed on male and female pups from 12 different dams according to the following protocol during pregnancy. In all cases animals were kept in a room with controlled temperature (22°C), a 12-h light, 12-h dark cycle (light on from 08:00 to 20:00 hours) and with free access to standard chow diet (unless specified) and water. Virgin female Wistar rats were mated with male rats (Charles River Laboratories, Barcelona, Spain). Day of conception (day 0 of pregnancy) was determined by examination of vaginal smears for the presence of sperm, and then female rats were single caged. Pregnant rats were divided into two groups (6 animals/group): one with free access to standard chow diet (3000 kcal/kg; Panlab, Barcelona, Spain), and the other one underwent a 20% restriction of caloric intake from day 1 to day 12 of pregnancy. Caloric restriction was performed by offering to each dam a daily amount of food corresponding to 80% of the calories that should be eaten according to their body weight. This amount was calculated considering the calories daily consumed by their control animals under *ad libitum* feeding conditions. After the caloric restriction period, rats were allowed to eat *ad libitum*, and food intake was measured. At day 1 after delivery, the number of pups per litter was registered. In this study, all litters had 10 or more pups. In order to keep 10 pups/dam (in an equal number of males and females, when possible), excess pups of each sex in each litter were removed in a randomized way. Weaning was conducted at 21 days of life, and animals were grouped in 2/cage.

On day 25 of life, animals from the control and caloric restricted (CR) groups were killed by decapitation under fed conditions, during the first 2 h of the beginning of the light cycle. Some of the animals (n = 5–7, per group) were used for DNA quantification and gene expression analysis and the others (n = 5, per group) to perform morphometric and immunohistochemical analysis in WAT.

A second set of animals (n = 5–8, per group) was kept alive and fed with standard diet until the age of 4 months; then they were exposed to a high fat (HF) diet (4.7 kcal/g, with 45% calories from fat, Research Diets, Inc., NJ, USA). HF diet contained 5.5% calories from soybean oil and 39.5% from lard. Body weight and food intake were measured weekly until the age of 6 months, when they were sacrificed.

Animals used for the different analysis were from at least four different litters. For gene expression studies and DNA measurements, iWAT and retroperitoneal WAT (rpWAT) were rapidly removed, weighed and immediately frozen in liquid nitrogen and stored at −70°C until RNA and DNA analysis. For morphometric analysis, samples of the afore-mentioned two WAT depots were fixed by immersion in 4% paraformaldehyde in 0.1 M phosphate buffer (pH = 7.4) at 4°C for 24 h, then washed and stored in 0.1 M phosphate buffer (pH = 7.4) until posterior analysis. The iWAT is a subcutaneous depot located at the base of the hind legs that extends in to the inguino-crural region (inguinal portion), and the rpWAT is an internal abdominal depot that lies in paravertebral position, on the border between the spine and the posterior abdominal wall [26].

### Real-time Quantitative PCR Analysis

Total RNA was extracted from WAT by Tripure Reagent (Roche Diagnostic Gmbh, Mannheim, Germany) according to the manufacturer's instructions. Isolated RNA was quantified using the NanoDrop ND-1000 spectrophotometer (NadroDrop Technologies, Wilmington, DE, USA) and its integrity confirmed using agarose gel electrophoresis. Real-time PCR was used to measure mRNA expression levels of β3-AR, α2-AR and leptin in WAT. 0.25 µg of total RNA (in a final volume of 5 µl) was denatured at 65°C for 10 min and then reverse transcribed to cDNA using MuLV reverse transcriptase (Applied Biosystem, Madrid, Spain) at 20°C for 15 min, 42°C for 30 min, with a final step of 5 min at 95°C in an Applied Biosystems 2720 Thermal Cycler (Applied Biosystem, Madrid, Spain). Each PCR was performed from diluted (1/5) cDNA template, forward and reverse primers (10 µM each), and Power SYBER Green PCR Master Mix (Applied Biosystems, CA, USA). Primer sequences and products for the different genes are described in table 1. All primers were obtained from Sigma (Madrid, Spain). Real-time PCR was performed using the Applied Biosystems StepOnePlusTM Real-Time PCR Systems (Applied Biosystems) with the following profile: 10 min at 95°C, followed by a total of 40 two-temperature cycles (15 s at 95°C and 1 min at 60°C). In order to verify the purity of the products, a melting curve was produced after each run according to the manufacturer's instructions. The threshold cycle (Ct) was calculated by the instrument's software (StepOne Software v2.0) and the relative expression of each mRNA was calculated as a percentage of control group, using the 2^−ΔΔCt^ method [27] and the more suitable reference gene for each case (18S, β-actin or GDP dissociation inhibitor 1 (GDI-1)).

**Table 1:** Nucleotide sequences of primers and amplicon size.

Gene	Forward Primer (5′ to 3′)	Reverse Primer (5′ to 3′)	Amplicon size (bp)
18S	CGCGGTTCTATTTTGTTGGT	AGTCGGCATCGTTTATGGTC	219
β-actin	TACAGCTTCACCACCACAGC	TCTCCAGGGAGGAAGAGGAT	120
GDI-1	CCGCACAAGGCAAATACATC	GACTCTCTGAACCGTCATCAA	210
β3AR	CCTTCAACCCGCTCATCTAC	TGGGAAATGGACGCTCAC	189
α2AR	GGTAAGGTGTGGTGCGAGAT	CAGCGCCCTTCTTCTCTATG	229
Leptin	TTCACACACGCAGTCGGTAT	AGGTCTCGCAGGTTCTCCAG	186

### Morphometric and Immunohistochemical Analysis

The two fixed WAT were dehydrated in graded series of ethanol, cleared in xylene and embedded in paraffin. Five-micrometer-thick sections of tissues were cut with a microtome and mounted in slides. Immunohistochemical demonstration of tyrosine hydroxylase (TH) and Neuropeptide Y (NPY) were performed with the avidin-biotin peroxidase (ABC) method [28]. In each assay, 11–12 samples of control and CR animals of a specific WAT depot, gender and age were processed. After rehydration, sections were incubated sequentially at room temperature in the following solutions: 0.3% hydrogen peroxide in methanol for 30 min to block endogenous peroxidase; 2% goat normal serum in Phosphate-buffered saline 0.05 M, pH = 7.4, 0.1% Triton X-100 (PBS) for 20 min to reduce non-specific background staining prior to incubation with primary antibody (polyclonal anti-TH antibody produced in sheep, AB1542, Chemicon International, 1∶750 in PBS with 1% BSA and polyclonal anti-NPY antibody produced in rabbit, N9528, Sigma-Aldrich, 1∶8000 in PBS with 1% BSA) overnight at 4°C; biotinylated goat anti-rabbit IgG and biotinylated rabbit anti-sheep IgG (Vector Laboratories, Burlingame, CA) 1∶200 in PBS 1% BSA for 30 min at room temperature; peroxidase-labeled ABC reagent (Vectastain ABC kit, Vector) in PBS for 1 h at room temperature and Fast 3,3′-diaminobezidine tablet, DAB (Sigma, St. Louis, MO, USA) in Tris for 5 min in dark room for enzymatic development of peroxidise. Subsequently, slides were washed with deionized water, stained with hematoxylin/eosin, dehydrated with increasing concentrations of ethanol and xylene, mounted with Eukitt (Panreac Quimica SA) and cover-slipped. Negative controls were performed by omission of primary antibody. For each animal, 16–20 inguinal and retroperitoneal WAT images from light microscopy random fields coming from two nonconsecutive sections (7–10 fields per section) were digitalized. Immunoreactive TH and NPY area (TH^+^ and NPY^+^ respectively) were measured interactively in each section using AxioVision40V 4.6.3.0. Software (Carl Zeiss, Imaging Solutios GmbH, Germany). The specific immunoreactive signal was recognized as the intensive brown colour usually observed near arterioles and capillaries. The software was also used to measure the diameter of adipocytes in two nonconsecutive hematoxylin/eosin stained sections (different from those in which immunohistichemistry was performed). On one random field taken from each section, the diameter 100 adipocytes were measured and averaged, and then, the media of both sections was calculated. Image analysis from all groups was examined in a blind fashion.

### Quantification of DNA levels

For quantification of DNA levels, 250–300 mg of WAT were homogenized in 3 volumes of PBS, and in the case of rpWAT of 25 day offspring, 60–70 mg in 9 volumes of PBS, using a polytron homogenizer, and then centrifuged at 500 g for 10 min; the supernatant was collected and used for DNA quantification by a fluorometric method that using 3,5 diaminobenzoic acid [29].

### Statistical Analysis

Data are expressed as means ± s.e.m. (n = 5–8). Student's t test was performed to assess differences between control and CR groups. Threshold of significance was defined at p<0.05.

## Results

### Body weight, and weights and morphological features of WAT depots

At the age of 25 d, male and female CR animals exhibited no significant differences in body weight or in the weight of different WAT depots with respect to control animals. Morphometric study also showed no significant changes in the diameter of adipocytes in the different depots, between control and CR animals, either in males or in females. Neither was there significant difference in the DNA expressed per g of tissue or in total DNA content in any of the analyzed depots between control and CR animals (Table 2).

**Table 2:** Weight and morphological features of WAT in 25 d and 6 m old animals.

			Males		Females	
			C	CR	C	CR
***25 d old***						
		Body weight (g)	63.2±3.3	59.3±2.0	60.4±1.8	55.7±2.8
	**iWAT**					
		Weight (mg)	632±69	684±49	689±98	739±53
		Adipocyte diameter (µm)	33.4±1.9	32.0±0.8	34.3±1.3	30.4±1.1
		DNA (µg/g wet tissue)	954±94	837±73	857±121	897±62
		Total DNA content (mg)	573±44	565±53	551±60	670±83
	**rpWAT**					
		Weight (mg)	113±12	95.7±7.8	95.4±16.5	92.2±18.4
		Adipocyte diameter (µm)	30.9±1.9	32.6±1.0	34.0±1.8	33.0±2.8
		DNA (µg/g wet tissue)	682±40	719±69	728±126	634±73
		Total DNA content (mg)	75.4±6.1	68.9±9.9	74.1±24.7	59.6±15.1
***6 m old***						
		Body weight (g)	487±9	548±21*	263±14	274±5
	**iWAT**					
		Weight (g)	11.7±1.1	16.3±1.6*	3.54±0.49	3.83±0.38
		Adipocyte diameter (µm)	71.6±2.6	71.8±3.3	54.2±2.6	58.4±3.0
		DNA (µg/g wet tissue)	188±30	176±9	301±48	286±60
		Total DNA content (mg)	1981±279	2825±221*	989±126	1023±210
	**rpWAT**					
		Weight (g)	15.3±1.1	19.0±1.7	2.96±0.34	4.43±0.61
		Adipocyte diameter (µm)	79.7±4.3	95.5±3.9*	76.6±4.1	75.6±3.5
		DNA (µg/g wet tissue)	123±3	97.6±11.3	73.7±5.1	70.0±11.1
		Total DNA content (mg)	1896±148	1823±236	223±36	285±35

Body weight and weight, adipocyte diameter, DNA expressed per gram of wet tissue and total DNA content of inguinal and retroperitoneal white adipose tissue depots (iWAT and rpWAT respectively), in 25 d and 6 m old male and female offspring of controls (C) and 20% caloric restricted dams during gestation (CR). Each group is made up of animals from at least four different litters. Data are mean ± s.e.m.

*Different from their respective C group (p<0.05; Student's t -test).

At 4 m of age, before exposition to HF diet, male CR animals, but not female CR, presented greater body weight compared with their respective controls (406±8 vs 437±6 p = 0.005 for control and CR males and 244±7 vs 243±2 p = 0.997 for control and CR female animals, respectively). The difference between control and CR male animals concerning body weight was even higher at the age of 6 m, after 2 m of HF diet feeding. (Table 2). This was associated with an increased weight of the iWAT depots in male CR animals, but not in females. In both male and female animals, the rpWAT of CR animals showed a tendency to greater weight compared with their controls, although the difference did not reach statistical significance (p = 0.10, and p = 0.08 for males and females, respectively; Student's t test).

In male animals, the increased weight of the iWAT compared with controls occurring at the age of 6 m was associated with a parallel increase in the total DNA content of the tissue. Nevertheless no differences were observed either in the diameter of adipocytes or in the DNA content expressed per g of tissue. This suggested that the greater adiposity in this depot is a consequence of the development of hyperplasia.

However, in the rpWAT, the adipocyte diameter of CR male animals was larger than that of their controls, while no differences were observed in total DNA content. These results suggest the development of hypertrophy in the rpWAT in male animals exposed to caloric restriction during gestation. In spite of the hypertrophy observed in this depot of CR male animals, the presence of crown-like structures, which are indicative of macrophage infiltration [30], was negligible at this age. No signs of macrophage infiltration were either observed in the iWAT.

No significant differences were found between control and CR female animals concerning morphometric parameters and DNA content, in any of the WAT depots studied (Table 2).

### Immunohistochemical analysis of TH and NPY in WAT depots

Immunohistochemical analysis of TH and NPY were performed in the inguinal and in the retroperitoneal depots at both ages.

**Figure 1:**
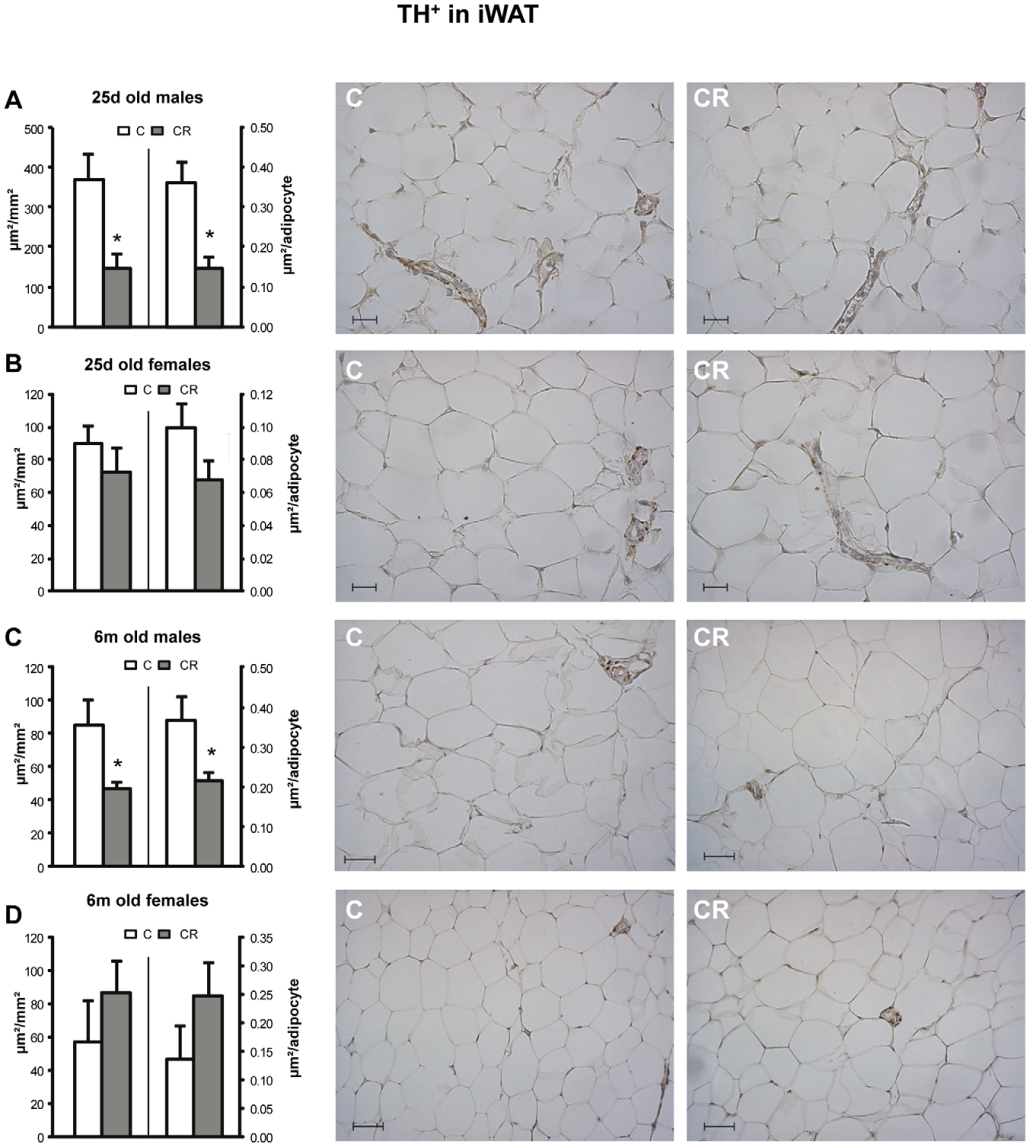
Immunohistochemical analysis of TH in iWAT depot. Immunoreactive TH area (TH^+^) - expressed as µm/mm^2^ (left axis) and as µm/adipocyte (right axis) - in inguinal white adipose tissue (iWAT) of 25 d (A and B) and 6 m (C and D), male (A and C) and female (B and D) offspring of controls (C) and 20% caloric restricted dams during gestation (CR) and their corresponding representative immunostained sections (C on the left and CR on the right). Scale bar: 20 µm and 50 µm for 25 d old and 6 m old animals respectively. Data are mean ± s.e.m. (n = 5–6, coming from at least four different litters). For each animal, 16–20 measurements were performed in different random fields coming from two non consecutive sections of the tissue (7–10 fields per section).* Different from their respective C group (p<0.05; Student's t -test).

Concerning TH, CR male animals showed a marked decrease in TH^+^ per mm^2^ of tissue in the iWAT compared with controls at both ages studied (25 d and 6 m), while no significant differences were found in CR female animals (Figure 1).

**Figure 2:**
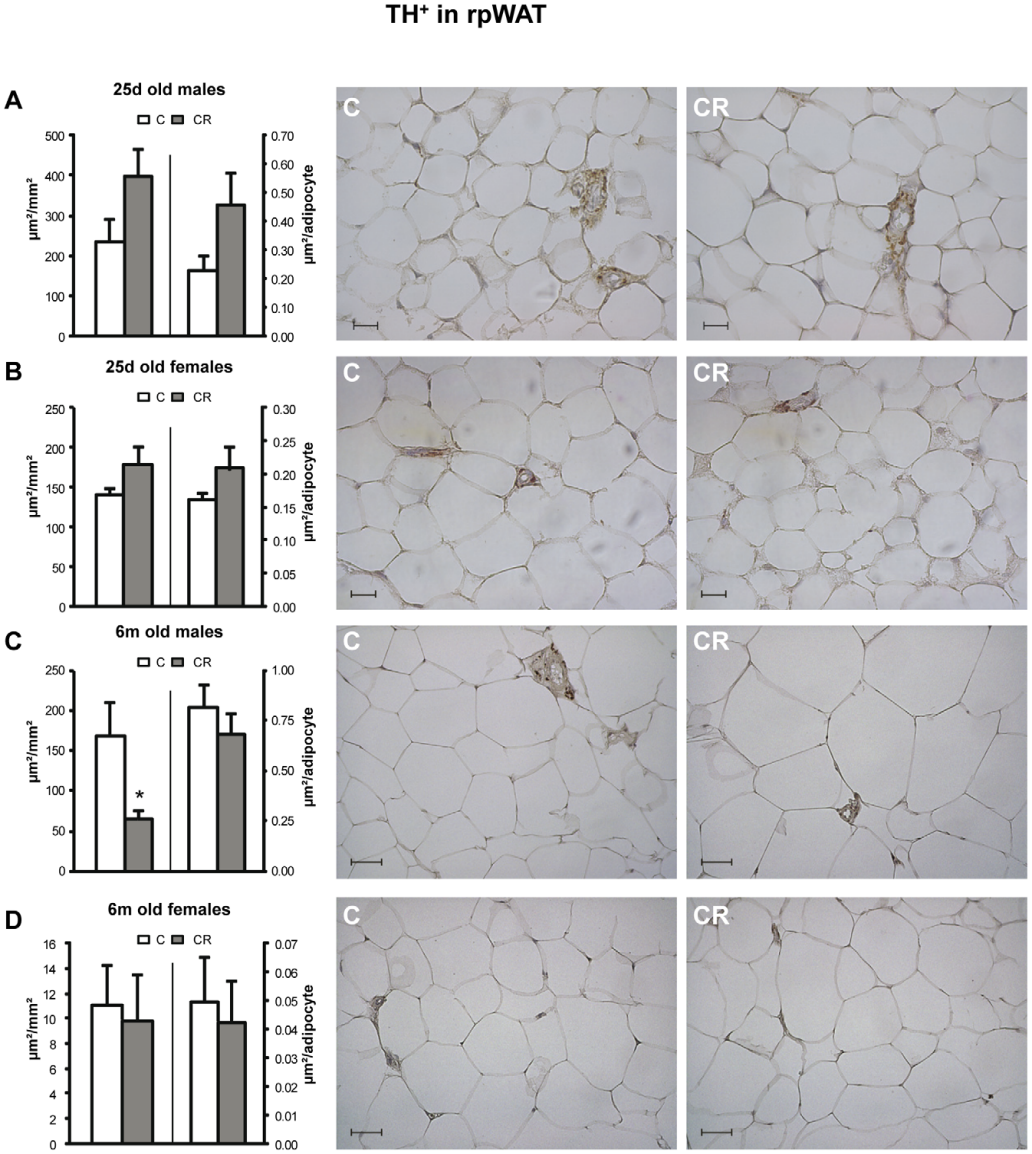
Immunohistochemical analysis of TH in rpWAT depot. Immunoreactive TH area (TH^+^) - expressed as µm/mm^2^ (left axis) and as µm/adipocyte (right axis) - in retroperitoneal white adipose (rpWAT) tissue of 25 d (A and B) and 6 m (C and D), male (A and C) and female (B and D) offspring of controls (C) and 20% caloric restricted dams during gestation (CR) and their corresponding representative immunostained sections (C on the left and CR on the right). Scale bar: 20 µm and 50 µm for 25 d old and 6 m old animals respectively. Data are mean ± s.e.m. (n = 5–6, coming from at least four different litters). For each animal, 16–20 measurements were performed in different random fields coming from two non consecutive sections of the tissue (7–10 fields per section).* Different from their respective C group (p<0.05; Student's t -test).

In the retroperitoneal depot, no significant differences were found between control and CR animals concerning TH^+^ per mm^2^ when they were 25 d old, whereas CR male animals, but not females, showed lower TH^+^ per mm^2^ than their controls when they were 6 m old (Figure 2).

**Figure 3:**
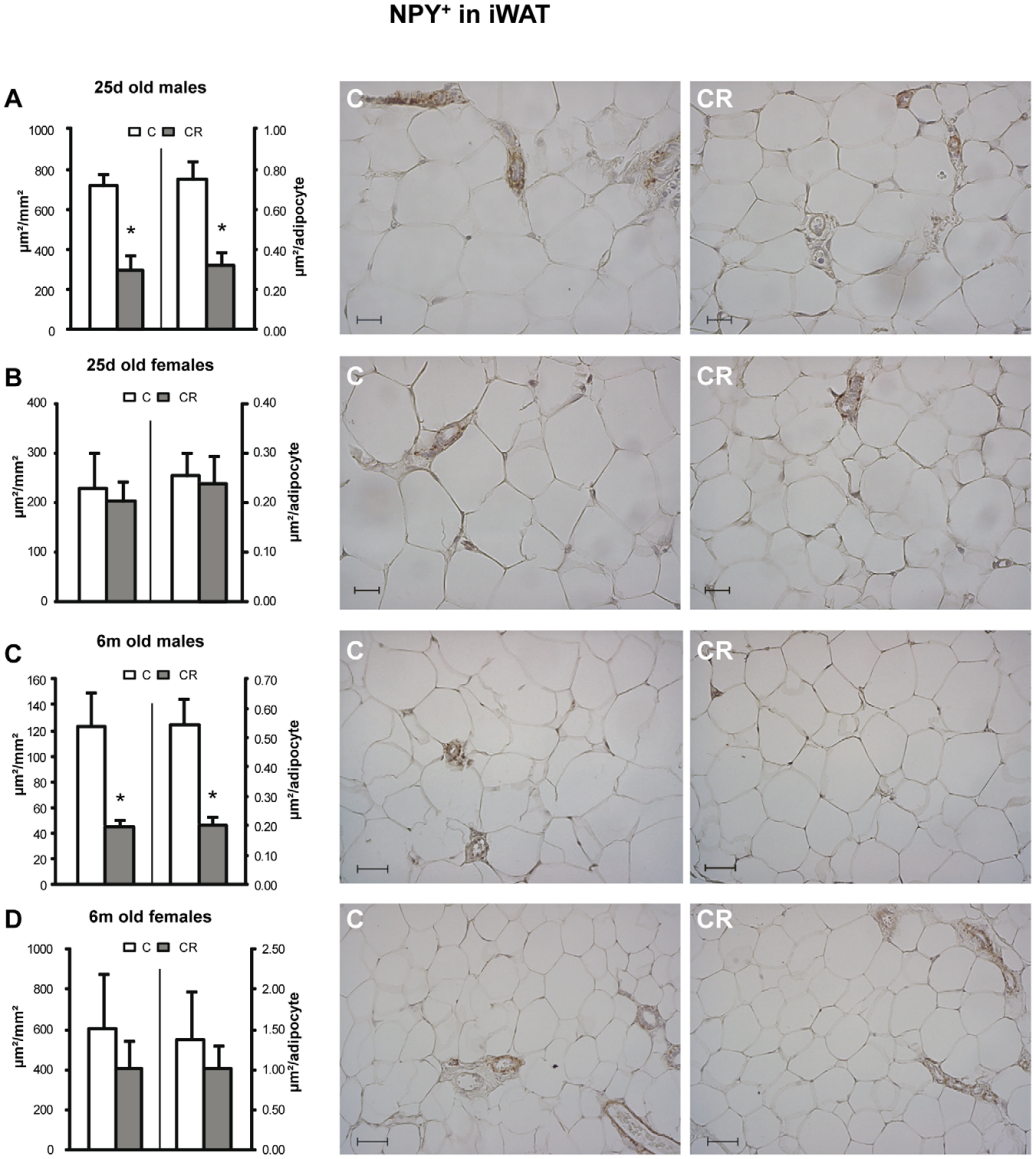
Immunohistochemical analysis of NPY in iWAT depot. Immunoreactive NPY area (NPY^+^) - expressed as µm/mm^2^ (left axis) and as µm/adipocyte (right axis) - in inguinal white adipose tissue (iWAT) of 25 d (A and B) and 6 m (C and D), male (A and C) and female (B and D) offspring of controls (C) and 20% caloric restricted dams during gestation (CR) and their corresponding representative immunostained sections (C on the left and CR on the right). Scale bar: 20 µm and 50 µm for 25 d old and 6 m old animals respectively. Data are mean ± s.e.m. (n = 5–6, coming from at least four different litters). For each animal, 16–20 measurements were performed in different random fields coming from two non consecutive sections of the tissue (7–10 fields per section).* Different from their respective C group (p<0.05; Student's t -test).

**Figure 4:**
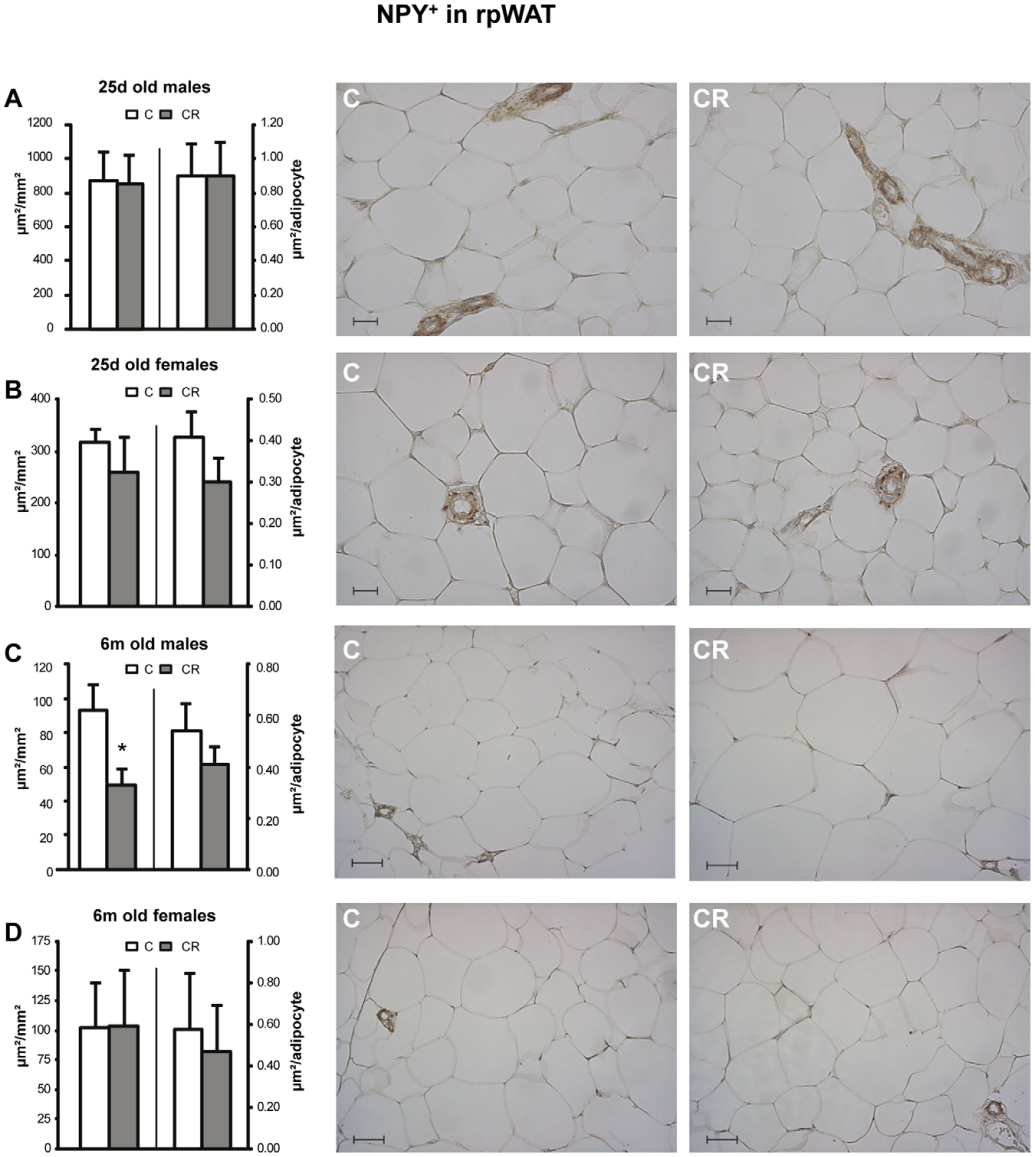
Immunohistochemical analysis of NPY in rpWAT depot. Immunoreactive NPY area (NPY^+^) - expressed as µm/mm^2^ (left axis) and as µm/adipocyte (right axis) - in retroperitoneal white adipose tissue (rpWAT) of 25 d (A and B) and 6 m (C and D), male (A and C) and female (B and D) offspring of controls (C) and 20% caloric restricted dams during gestation (CR) and their corresponding representative immunostained sections (C on the left and CR on the right). Scale bar: 20 µm and 50 µm for 25 d old and 6 m old animals respectively. Data are mean ± s.e.m. (n = 5–6, coming from at least four different litters). For each animal, 16–20 measurements were performed in different random fields coming from two non consecutive sections of the tissue (7–10 fields per section).* Different from their respective C group (p<0.05; Student's t -test).

A very similar pattern to that of TH was found concerning NPY immunoreactive area NPY^+^ per mm^2^. CR male animals showed lower NPY^+^ per mm^2^ than their controls at both ages studied in the iWAT (Figure 3). In the rpWAT, CR males showed lower immunoreactive area when they were 6 m old, but not when they were 25 d old (Figure 4). Again, no significant differences were found between control and CR female animals, either in the inguinal or in the retroperitoneal depots (Figures 3 and 4).

### mRNA expression levels of β3-AR and α2-AR in WAT depots

To check whether changes in sympathetic innervation of adipose tissue between control and CR animals may affect NE signalling in this tissue, mRNA expression levels of β3-AR and α2-AR were measured in both adipose tissue depots (Table 3).

**Table 3:** mRNA expression levels of β3-AR and α-2AR in WAT in 25 d and 6 m old animals.

			Males		Females	
			C	CR	C	CR
***25 d old***						
	**iWAT**					
		β3-AR (%)	100±8	129±10*	100±14	84.1±8.6
		α2-AR (%)	100±11	56.3±14.7*	100±15	103±17
	**rpWAT**					
		β3-AR (%)	100±10	91.0±7.3	100±13	67.1±5.0*
		α2-AR (%)	100±16	97.1±8.7	100±32	64.3±8.9
***6 m old***						
	**iWAT**					
		β3-AR (%)	100±12	118±26	100±17	146±18
		α2-AR (%)	100±18	35.9±5.7*	100±14	155±29
	**rpWAT**					
		β3-AR (%)	100±22	93.6±14.3	100±18	118±18
		α2-AR (%)	100±17	66.1±24.0	100±13	155±38

mRNA expression levels of β3 and α2 adrenergic receptor (β3-AR and α2-AR) in inguinal and retroperitoneal white adipose tissue depots (iWAT and rpWAT respectively), in 25 d and 6 m old male and female offspring of controls (C) and 20% caloric restricted dams during gestation (CR). mRNA expression levels in male and female controls are set to 100% and values of CR animals expressed relative to their controls. Each group is made up of animals from at least four different litters. Data are mean ± s.e.m.

*Different from their respective C group (p<0.05; Student's t -test).

At the age of 25 d, CR male animals showed higher mRNA expression levels of β3-AR and lower α2-AR in the iWAT compared with controls. The lower expression levels of α2-AR persisted in adulthood in CR animals, while expression of β3-AR was not different than controls at the age of 6 m. These changes did not occur in the retroperitoneal depot, which did not show evidence of lower SNS innervation.

No significant changes in mRNA expression levels of β3-AR and α2-AR occurred between control and CR female animals in the inguinal depot, in accordance with the lack of effects of these particular conditions during gestation on sympathetic innervation in these animals. However, in the rpWAT, CR female animals showed lower β3-AR mRNA levels than their controls at the age 25 d, although this was accompanied by a tendency to lower α2-AR mRNA levels, and hence the β3-AR/α2-AR ratio was not significantly affected (data not shown).

### Leptin mRNA expression levels in WAT depots

Leptin mRNA expression levels were measured in the inguinal and retroperitoneal fat depots of male and female control and CR animals at both ages (25 d and 6 m) (Table 4).

**Table 4:** mRNA expression levels of Leptin in WAT in 25 d and 6 m old animals.

		Males		Females	
		C	CR	C	CR
***25 d old***					
	iWAT Leptin (%)	100±8	108±6	100±11	85.3±7.9
	rpWAT Leptin (%)	100±13	81.9±8.5	100±18	69.7±6.3
***6 m old***					
	iWAT Leptin (%)	100±28	192±29*	100±44	105±30
	rpWAT Leptin (%)	100±34	89.2±24.2	100±42	37.9±14.3

mRNA expression levels of Leptin in inguinal and retroperitoneal white adipose tissue depots (iWAT and rpWAT respectively), in 25 d and 6 m old male and female offspring of controls (C) and 20% caloric restricted dams during gestation (CR). mRNA expression levels in male and female controls are set to 100% and values of CR animals expressed relative to their controls. Each group is made up of animals from at least four different litters. Data are mean ± s.e.m.

*Different from their respective C group (p<0.05; Student's t -test).

No significant differences were found between control and CR animals concerning leptin expression when animals were 25 d old, either in male or female animals. When animals were 6 m old, CR male animals showed higher leptin mRNA expression levels in the iWAT compared with controls, with no changes in the rpWAT. No differences were found between control and CR female animals in either depot studied concerning leptin mRNA expression levels.

## Discussion

Here we show for the first time that moderate caloric restriction during the first part of pregnancy leads to a reduced noradrenergic innervation of the subcutaneous WAT in the offspring, suggesting that nutrition during gestation can modulate peripheral nervous system development. We also show that the effects produced by this caloric restriction on adipose tissue innervation are gender-dependent, since they are evident in males but not in females. It is suggested that alterations occurring in the development of SNS may be one of the mechanisms responsible for the lasting effects of caloric restriction during gestation on later adiposity of the offspring, which also occurs particularly in male animals, but not in females, and in a depot-dependent manner. In particular, the hyperplasia occurring in the iWAT of adult males exposed to a HF diet may be explained by a reduced innervation in this adipose depot occurring as a consequence of malnutrition during a critical period of development.

Increased adipose tissue mass is the primary phenotypic characteristic of obesity. Under conditions of a positive energy balance, it is known that the deposition of lipid initially results in increased fat cell size, but soon triggers increases in fat cell number [31]. However, the processes underlying the hypercellularity in the adipose tissue are not well understood (for review, see Ref. [32]). Thus research into factors and molecular mechanisms influencing adipose cell proliferation and differentiation, and the conditions that favour these processes in a depot specific manner, represent a crucial area of research.

Moderate caloric restriction in rats during the first half of gestation has been previously described to have lasting effect in the offspring, programming animals for greater food intake [6]. We observed that in male animals this results in greater body weight gain and increased body weight in adulthood, even under normal fat diet conditions [6]. However, female animals seem to be protected against fat accumulation in adult life [6]. Another study performed by exposing pregnant rats to more severe caloric restriction (50%) also showed that the male offspring became hyperphagic and gained more weight than controls, while females did not overeat and did not became obese [4].

Here, we also observed that moderate caloric restriction during the first half of gestation had no apparent effects on body weight and adiposity in animals at a juvenile age (25 d old) but did result in significant effects on body weight and adiposity in adult male animals, but not in females. In particular, at the age of 6 m, CR male animals exhibited 12.5% excess of body weight with respect to their controls, which was associated with increased fat accumulation, although with depot-specific differences. The iWAT depot showed 39.3% greater weight than their controls, and the excess of fat was associated with an increased number of adipocytes, as suggested by the increased total DNA content in the tissue (42.6% increase), without showing differences in the adipocyte size. Thus, although adipocyte proliferation was not directly assessed, these results suggest the development of hyperplasia in the inguinal depot of adult male animals as a consequence of caloric restriction during gestation. On the other hand, the significant increase in the adipocyte size of CR male animals in the retroperitoneal depot (19.8% increase) compared with controls, with no changes in total DNA content of the tissue, suggests the development of hypertrophy in this depot.

The role of SNS in the adipose tissue on the regulation of lipid mobilization is well established [11], [12]. However, a more recent role of SNS innervation of WAT has been described in the control of adipose tissue growth and cellularity [15]. Specifically, NE, the main sympathetic postganglionic neurotransmitter, inhibits the natural proliferation of adipocytes in culture [19]. Furthermore, denervation of WAT triggers impressive hypercellularity in Siberian hamsters [33] and in laboratory rats [17], providing *in vivo* support for the role of the SNS/NE in the control of fat cell number. Thus, the level of noradrenergic innervation in the adipose tissue could determine the degree of adipocyte proliferation and the posterior development of hyperplasia in the tissue.

Sympathetic innervation in both the inguinal and retroperitoneal depot was determined by assessing tissue staining for TH, which is the rate-limiting enzyme for catecholamine synthesis [34]. Of interest, in CR males, noradrenergic innervation of iWAT, but not in rWAT, was significantly reduced at early life, so the adrenergic drive to the subcutaneus depot may be deficient, a situation that could resemble partial dennervation and could hence explain the development of hyperplasia in iWAT in adult CR males. The idea of partial dennervation is not as strange if we consider previous studies showing that 50% undernutrition during the last two weeks of pregnancy can reduce the number of prevertebral sympathetic neurons, and also reduce the enteric sympathetic innervation in the offspring [10], [9]. Moreover, other studies have shown that a protein deficient diet (5%) during gestation and after produces atrophy and neuron loss in sympathetic ganglion neurons of rat offspring [35]. Thus, it is feasible that caloric restriction during gestation could lead to partial noradrenergic dennervation of the inguinal adipose tissue and therefore favour the hyperplasia seen in this fat depot in adulthood; however more functional studies could be performed to fully demonstrate a true impairment in sympathetic regulation.

In adulthood, at the age of 6 months, noradrenergic innervation of iWAT of CR males was also reduced, but this alteration also occurred in the rpWAT. Nevertheless, in the latter, this may be attributed to the increased size of adipocytes, since the decrease was not significant when TH^+^ was referred to the adipocyte number. However, it must be mentioned that since at 6 months of age these animals were under HF diet, it is difficult to distinguish whether the reduced noradrenergic innervation is only a consequence of maternal caloric restriction or it is also an interaction of this condition with HF diet exposure and even with the effects of age.

Unlike males, changes in SNS innervation in iWAT or rWAT were not apparent in female animals, neither were there changes in fat accumulation in these depots, as mentioned above, even under conditions that promote body fat accretion. Thus, female animals seem to be more resistant to the negative effects associated to caloric restriction during gestation, as previously described [6]. Female rats have also been described to be more resistant to obesity-linked disorders associated to HF diet exposure [36].

To substantiate the results obtained for TH, we also performed immunohistochemical analysis for NPY in both adipose tissue depots, since this peptide is known to be colocalized with NE and released together with NE upon sympathetic activation [22]. Results concerning NPY^+^ were very similar to those obtained for TH, evidencing a decrease in sympathetic innervation in the iWAT of CR male animals but not in the rpWAT. NPY seems to exert an opposite function to that of NE, since it promotes proliferation [24]. Thus, although NPY levels in the nerve terminals have not been directly measured, the lower immunostainning for NPY in the nerve terminals of the iWAT of CR male animals compared with control does not agree with increased cellularity. In accordance with our results, sympathetic denervation of WAT in hamsters also leads to increased adiposity and fat cell number than controls [18], [16], [33]. This has been explained by the removal of the inhibitory effect of NE on adipocyte proliferation [18]; therefore suggesting that the effects of NE may be dominant over that of NPY within the sympathetic nerve system of WAT, as previously pointed out [24].

The mechanisms underlying the inhibition of cell proliferation by NE are not well known beyond the activation of β-AR, particularly β3, and the resulting increase in cAMP [19], [20]. The α-AR, particularly the α2 subtype may also play a role, acting in an opposite way to that of β3-AR, since activation of α2-AR has been described to promote preadipocyte proliferation in culture [37]. NE stimulation has been described to reduce β3-AR levels in brown adipocytes [38]. Here the higher mRNA expression levels of β3-AR and lower α2-AR (and then higher β3/α2 ratio) in the iWAT of 25 d old male CR animals compared with controls may be the result of a compensatory mechanism due to the decreased NE stimulation of these adipocytes. In fact, these changes do not occur in the retroperitoneal depot, which does not show evidence of lower SNS innervation. Lower expression levels of α2-AR in the iWAT of CR male animals persisted in adulthood, while expression of β3-AR was not different from controls.

The aforementioned different effects on internal and subcutaneous fat depots of male offspring may be expected to affect the production of adipokines involved in energy homeostasis, such as leptin. Of interest, increased body weight occurring in CR male adult animals was associated with increased leptin expression in the iWAT, but not in the rpWAT. Leptin expression is known to be influenced by the status of fat stores [39], as well as by adipocyte size, as larger adipocytes produce more leptin than smaller adipocytes in the same individual [40]. However, regional-specific differences in the ontogenic pattern of leptin production within distinct depots of WAT have been described [41]. The rpWAT, which is a main contributor to circulating leptin in rats, increases leptin mRNA expression levels with age, in parallel to the increase in fat accumulation, but attains maximum levels in adult animals (when animals are about 7 m old) [41]. On the other hand, the iWAT steadily increases leptin expression during development, without attaining the maximal levels observed in the rpWAT [41]. This suggests that adipocytes from the iWAT may maintain their capacity to increase leptin mRNA expression in response to an additional accumulation of lipids, and thus explain the increased expression occurring in the iWAT, but not in the rpWAT, of CR males. Moreover, considering the inhibitory effect of NE on leptin expression by the adipocyte [42], we cannot rule out that the lower sympathetic innervation occurring in the iWAT of CR male animals could contribute to the increased leptin mRNA expression found in this depot in adult animals; however this aspect has not been directly addressed in this study and needs further research.

The mechanisms underlying the differential effects of caloric restriction during gestation on the inguinal and retroperitoneal depots in male animals are not known. However, it must be considered that although the SNS outflow from brain to WAT appears to be similar among rpWAT and iWAT, as described in Siberian hamsters [18], some apparent differences in the pattern of the central origins of this innervation have been described. rpWAT appears to have more marked innervation from the suprachiasmatic nucleus of the hypothalamus and from the nucleus of the solitary tract in the brain stem than does iWAT [43]. Therefore, the potential exists for separate control by the CNS of the SNS drive on WAT at these distal levels of the neuroaxis and thus a possible neuroanatomical basis for the differential programming effects of perinatal conditions as well as on the inhibition of WAT cell proliferation. Moreover, at least in Siberian hamsters, the inguinal depot seems to be more prone to develop hyperplasia than the retroperitoneal one [18].

All in all, these results, together with our previous studies [7] point out that early effects of caloric restriction during gestation on later obesity development may be related with abnormal development of central structures involved in food intake and energy expenditure control, further showing that differences in the adult phenotype of different adipose tissue depots may also be associated with gender and fat depot specific alterations in sympathetic innervation which may have an impact on WAT architecture. The function of sympathetic innervation of WAT as an inhibitor of fat cell number via inhibition of fat cell proliferation is of interest, both for a more complete understanding of WAT growth and for understanding the pathology of adiposity, given that increased fat cell proliferation and the subsequent hypercellularity are a hallmark of obesity.

In summary, moderate maternal caloric restriction (20%) during the first half of gestation results, in the adult offspring, in greater body weight and adiposity in males but not in females. The accumulation of the excess of fat is due to hyperplasia in the inguinal adipose tissue, which seems to be associated with reduced sympathetic innervation of the tissue, and hypertrophy in the retroperitoneal adipose tissue, with no significant effects in female animals. Thus, these results suggest that the different outcomes of maternal caloric restriction on male and female offspring on later adiposity can be explained, at least in part, by the effects of this perinatal condition on SNS development. The mechanisms underlying the different outcomes in males and females need further research.
